# A Hierarchical Siamese Network for Noninvasive Staging of Liver Fibrosis Based on US Image Pairs of the Liver and Spleen

**DOI:** 10.3390/s23125450

**Published:** 2023-06-08

**Authors:** Xue Wang, Ling Song, Yan Zhuang, Lin Han, Ke Chen, Jiangli Lin, Yan Luo

**Affiliations:** 1 College of Biomedical Engineering, Sichuan University, Chengdu 610065, China; 2 Department of Ultrasound, West China Hospital, Sichuan University, Chengdu 610065, China

**Keywords:** liver fibrosis, Siamese network, liver–spleen texture comparison, US images

## Abstract

Due to the heterogeneity of ultrasound (US) images and the indeterminate US texture of liver fibrosis (LF), automatic evaluation of LF based on US images is still challenging. Thus, this study aimed to propose a hierarchical Siamese network that combines the information from liver and spleen US images to improve the accuracy of LF grading. There were two stages in the proposed method. In stage one, a dual-channel Siamese network was trained to extract features from paired liver and spleen patches that were cropped from US images to avoid vascular interferences. Subsequently, the L1 distance was used to quantify the liver–spleen differences (LSDs). In stage two, the pretrained weights from stage one were transferred into the Siamese feature extractor of the LF staging model, and a classifier was trained using the fusion of the liver and LSD features for LF staging. This study was retrospectively conducted on US images of 286 patients with histologically proven liver fibrosis stages. Our method achieved a precision and sensitivity of 93.92% and 91.65%, respectively, for cirrhosis (S4) diagnosis, which is about 8% higher than that of the baseline model. The accuracy of the advanced fibrosis (≥S3) diagnosis and the multi-staging of fibrosis (≤S2 vs. S3 vs. S4) both improved about 5% to reach 90.40% and 83.93%, respectively. This study proposed a novel method that combined hepatic and splenic US images and improved the accuracy of LF staging, which indicates the great potential of liver–spleen texture comparison in noninvasive assessment of LF based on US images.

## 1. Introduction

### 1.1. Background

Liver fibrosis (LF) results from chronic damage to the liver in conjunction with the accumulation of extracellular matrix (ECM) proteins [1]. It is a wound-healing response to chronic liver injury, and the sustained buildup of collagens distorts the liver parenchyma and vascular architecture, resulting in impaired liver function and scar deposition [2]. All kinds of chronic liver diseases (CLDs) can lead to LF, and it may progress into cirrhosis without timely treatment [3]. Advanced LF and cirrhosis are associated with some life-threatening diseases such as hepatocellular carcinoma (HCC) and liver failure [4]. Therefore, accurate fibrosis staging is important for clinicians to make proper treatment and management decisions.

The “gold standard” for diagnosing LF is liver biopsy, which can provide useful information about the progress of liver disease and the formation of fibrosis [5,6]. However, liver biopsy is invasive, and the results may be influenced by sampling errors and interpretative variations [7,8,9]. The development of imaging diagnosis has evolved as an attractive alternative to liver biopsy. MRI and CT are routinely used to assess cirrhosis and its complications [10,11]. However, CT imaging is radiative and MRI imaging often requires expensive equipment. Moreover, both modalities are costly and time-consuming, and the discrimination of early and advanced stages of fibrosis remains challenging [12]. Compared with CT and MRI, US imaging has the advantage of being nonradiative and relatively inexpensive. US is able to image a large liver volume to reduce sampling errors and has the capacity to evaluate the echotexture of the liver parenchyma and the liver masses. With the formation of fibrous tissue, changes in the liver such as inhomogeneous parenchyma, a coarse echo pattern and a nodular liver surface can be observed with US imaging [13]. Despite the advantages of ultrasound, US-image-based LF staging is still disputed [14]. In clinical practice, experienced US specialists can roughly determine the degree of LF in patients based on the comprehensive ultrasonic manifestations, but the interpretation of US images is difficult and subjective, and due to variations in the quality of the US images, the diagnostic consistency is poor. Therefore, it is necessary to develop an objective and accurate method for LF staging with US images.

**Figure 1:**
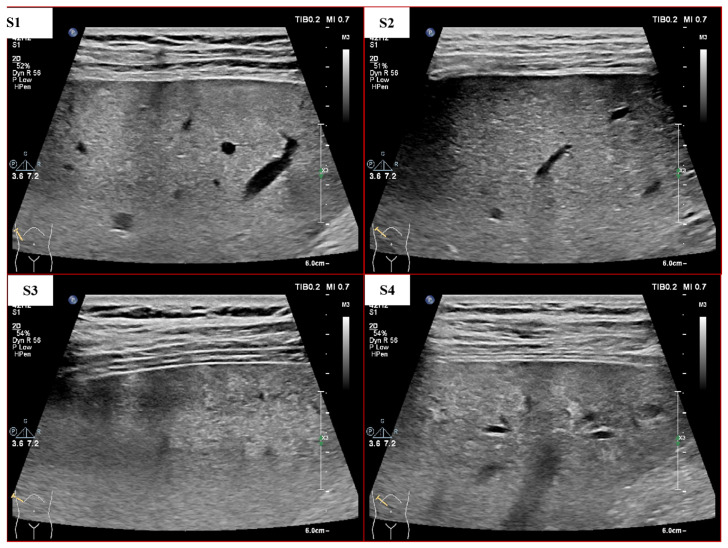
US images with different degrees of LF (S1–S4 means liver fibrosis stages 1–4, which are determined through liver biopsy by pathologists).

Although many LF staging methods based on machine learning (ML) have been proposed, the automated grading of LF remains challenging due to the slight texture differences between different LF stages, as shown in Figure 1. In addition, the quality of US images is strongly influenced by equipment performance, operator skills, noise and individual differences between patients [15]. Therefore, the performance of automated diagnosis methods still needs to be improved.

### 1.2. Related Works

The noninvasive diagnosis of liver fibrosis is mainly based on serum markers, CT, MRI and US examinations. In the studies based on US examinations, echotextures of the liver parenchyma are important diagnostic bases. LF is a kind of diffuse liver disease, and there may be multiple lesions with uneven distributions on the US image. There are works that considered the whole original image as research data without specifying an ROI [16,17,18]; instead, researchers utilized the original image by manually extracting information of interest [17,19] or combined it with other clinical parameters [18], which means that prior knowledge or data other than US images will be required while using these methods for LF staging. Most studies selected ROIs from the original images as objects of research, and manually outlining an ROI is the most common method of ROI selection [20,21,22,23]. A study used two morphological image operations (dilation and erosion) to remove the noise and join disparate meaningful areas with continuous contours as the ROI of an image [24]; the selected ROI preserved all parenchyma areas and eliminated interference from parameters, body postures and other text information in the background, but blood vessels were involved. Saito et al. used a U-Net to segment the liver parenchymal region and extracted ROIs with a size of 128 × 128 by sliding 12 pixels in the horizontal and vertical directions on the segmented area [25]. This method avoids blood vessels, but some blurred or useless areas may be included in the ROIs.

At present, most of the studies based on US images are retrospective, and there are the common problems of a small and imbalanced dataset. Transfer learning (TL) is introduced in many studies to overcome the problem of a small dataset to a certain extent, and for the imbalance of data distribution, researchers merge data into clinically meaningful classes such as significant fibrosis (≥S2) and advanced fibrosis (≥S3). Meng et al. [20] proposed a deep learning classification model composed of a VGGNet and a fully connected network (FCNet) and introduced TL to classify normal, early-stage fibrosis (S1–S3) and late-stage fibrosis (S4) based on 279 liver US images, and the accuracy was 63.28%. A study summarized 13 key points of liver fibrosis diagnosis based on prior knowledge from references and doctors and collected 10 US images indicating these 13 key points for each patient to train a multi-indicator-guided DL model for LF diagnosis. The method achieved an accuracy of 65.6% based on 229 patients [19]. Ruan et al. [26] proposed a multi-scale texture network based on 508 patients’ liver US images for a significant fibrosis (≥S2) diagnosis and achieved a sensitivity of 85.1%. Lee et al. [27] used a big dataset containing 13,608 US images from 3446 patients to train a DCNN for fibrosis staging. The accuracy of multi-classification (F0 vs. F1 vs. F23 vs. F4) in this study reached 83.5%. Most DL- and US-image-based LF staging methods are conducted with limited data, and the accuracy needs to be improved. Based on a small dataset, some researchers manually extracted texture features from US images and trained DL or ML models for LF staging. Different features were extracted in different studies, Li et al. achieved an accuracy of 80.09% for fibrosis diagnoses with MFLCM and WMCM features [16]; however, Xie et al. achieved an accuracy of 95.29% with 11 image features and the patient’s age and gender [17]. Research based on manual feature extraction requires a complex and subjective feature selection process, and the performance of the LF staging changes with the selected features. Some studies combined the US liver image with clinical parameters and other testing results to diagnose LF. Liu et al. used US images, liver stiffness values and the demographic and serological parameters of patients with hepatitis B to train a DL model for significant fibrosis recognition and achieved an AUC of 0.901 [18]. This model shows a good result for LF recognition, but it is not easy to collect all the required parameters of the patients, leading to a low diagnostic efficiency. Aiming to realize simple and rapid automated LF screening and monitoring, the performance of US-image-based models needs to be improved. 

In this study, a hierarchical Siamese network was proposed for automated LF staging based on both hepatic and splenic US images. First, we introduced a patch-based image processing workflow to extract patches from an ROI selected by doctors, which efficiently reduced the interference of blood vessels and the background in ultrasound images. In stage one, we trained a dual-channel Siamese network to extract both hepatic and splenic features from paired liver and spleen patches and quantified the liver–spleen difference (LSD) with the L1 distance. The LSD values were analyzed using kernel density estimation (KDE) to show the correlation between the LSD and the LF degree, which is rarely discussed in related works. In stage two, the weights pretrained in stage one were transferred into the Siamese feature extractor of the LF staging model, which was trained on the fusion of the liver and LSD features to improve the accuracy of LF staging. The proposed method showed great performance in liver cirrhosis (S4) diagnosis, advanced fibrosis diagnosis (≥S3) and multi-staging of fibrosis (≤S2, S3, S4). Compared with the general DL classification models that are trained with liver US images, the precision and sensitivity of our method for cirrhosis diagnosis were improved by about 8%, and the accuracies of advanced fibrosis diagnosis and multi-staging were both improved by about 5%, which indicated the great potential of liver–spleen texture comparison in noninvasive assessment of LF based on US images.

## 2. Materials and Methods

### 2.1. Data Acquisition

All data used in this study were collected from the West China Hospital of Sichuan University with the informed consent of the patients and passed the ethical review. A total of 286 patients were included in the study, and we collected ultrasound image pairs of the liver and spleen for each patient. All cases in this dataset had different degrees of liver fibrosis due to hepatitis B and received hepatectomies within 15 days after ultrasonography. A liver biopsy was performed after the hepatectomy to obtain the fibrosis stage of each case as a ground truth. In this study, we invited pathologists to stage liver fibrosis according to the Scheuer scoring system based on the pathological specimen. Table 1 shows the demographics of the patients and the clinical description of Scheuer scoring system for each stage [28].

### 2.2. Ultrasound Image Signs of Liver Fibrosis

CLDs that cause liver fibrosis and cirrhosis during the compensatory period often have no obvious clinical symptoms, leading to an untimely diagnosis. However, the liver structure, blood vessels and liver parenchyma will have various changes due to the formation of fibrosis in the wound-healing process of CLD. The specific signs of LF can be observed through US imaging. Table 2 shows the corresponding relationship between the US image signs and the stages of LF in patients with hepatitis B, in which the stages of LF are determined according to the pathological Scheuer scoring system [29].

The US image signs of the liver and spleen parenchyma in the same person should be similar under normal circumstances. With the progress of LF, the US examination of the liver shows an inhomogeneous echo and a rough liver parenchyma surface due to the proliferation of hepatic fibrous tissue, and generally, the higher the degree of liver fibrosis, the more obvious the US image signs. Meanwhile, the echotexture of the spleen parenchyma will not change significantly, which makes the US image signs of the liver and spleen in the same patient present different texture features. A study showed that adding CT-based splenic radiomic features to hepatic radiomic features increases radiomic analysis performance for LF staging [30], but there are few reports of US splenic texture features used for US-image-based LF staging. 

**Figure 2:**
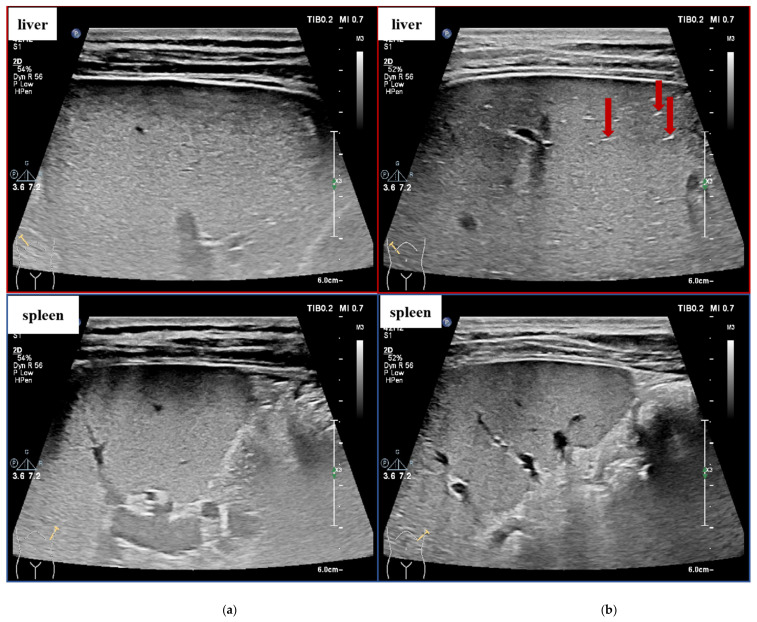

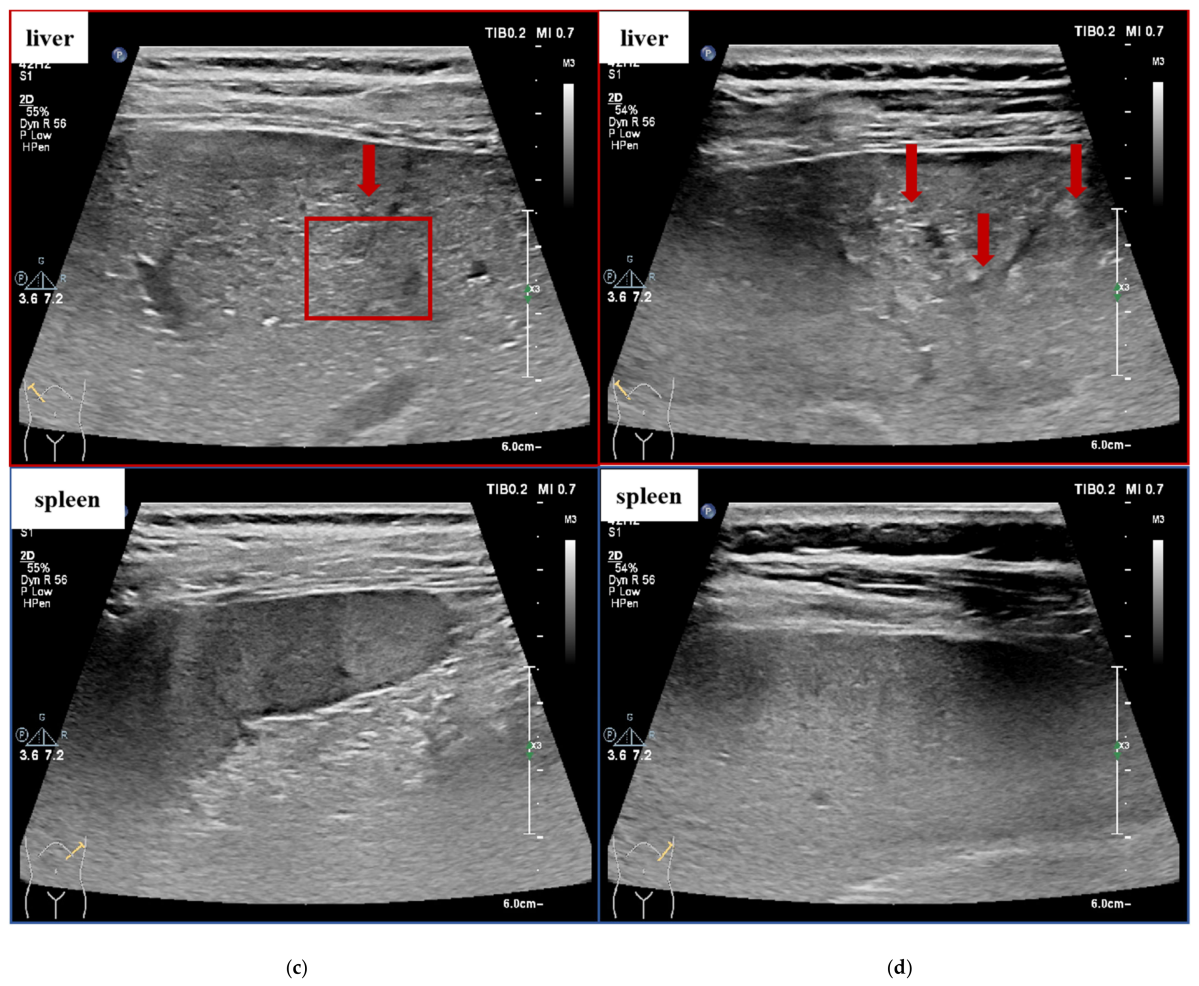
US image signs of fibrotic liver and spleen (S1–S4 means liver fibrosis stages 1–4, which are determined through liver biopsy by pathologists. The red arrows pointing to US signs of fibrosis as examples). (**a**) S1: Rough echo of liver parenchyma. (**b**) S2: “strip pattern” liver fibrosis. (**c**) S3: significantly rough echo of liver parenchyma with uneven distribution. (**d**) S4: hyperplasia nodules of liver parenchyma.

Figure 2 shows some example cases in our dataset. Liver images with different LF stages show possible US image signs, and it is more obvious to see the changes in the liver parenchyma when comparing them with the spleen US images from the same patient. In this study, we proposed a two-stage Siamese network to analyze the correlation between the stage of fibrosis and the liver–spleen differences (LSDs) and combined the LSDs for LF staging to improve the performance of automated LF staging models.

### 2.3. Patch-Based Image Processing

**Figure 3:**
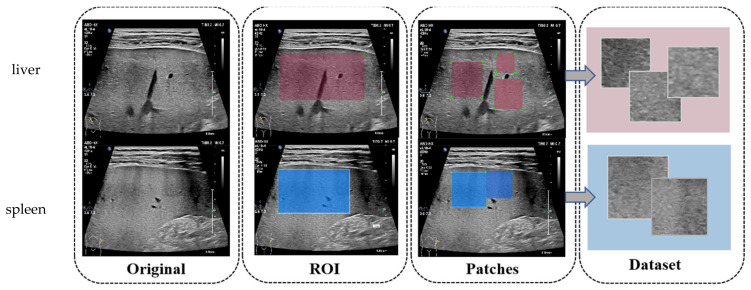
The workflow of image processing.

The original US liver and spleen images contain large areas of useless information, so the first step of image processing is the selection of the area of interest (ROI) by an US doctor. The selected ROI showed the observation area of the liver and spleen parenchyma and excluded the fuzzy and obvious noise interference in the original images. Based on the ROI, we cropped the image into patches to avoid the interferences of vascularity, because there are abundant and irregular vascular manifestations in US images of the liver, which will affect the automated feature extraction of LF. The principles of patch selection were as follows: (1) All patches should be within the ROI selected by the doctors, with at most 20 pixels of boundary overflow if necessary. (2) The aspect ratio of the patches should be as close as possible to 1:1 to avoid a resize operation affecting texture patterns. (3) The size of patch is not fixed but should be greater than 100 × 100, and the liver and spleen patches do not have to be the same size. (4) The overlapping area between patches from the same ROI should not exceed 10%. Under these principles, 1~6 patches can be cropped from one ROI. After patch cropping, there were a total of 4553 pairs of images and 2–30 pairs for each case. Figure 3 shows the workflow of image processing. The echotextures in patches from the same patient were similar, so we labeled every patch the same grade of fibrosis as the original image. Patch cropping augmented the small dataset used in our study and effectively alleviated the problem of overfitting. The images were grouped by patient; 226 patients were used for training and 60 patients were held out for independent validation.

### 2.4. Two-Stage Siamese Network for LF Staging

**Figure 4:**
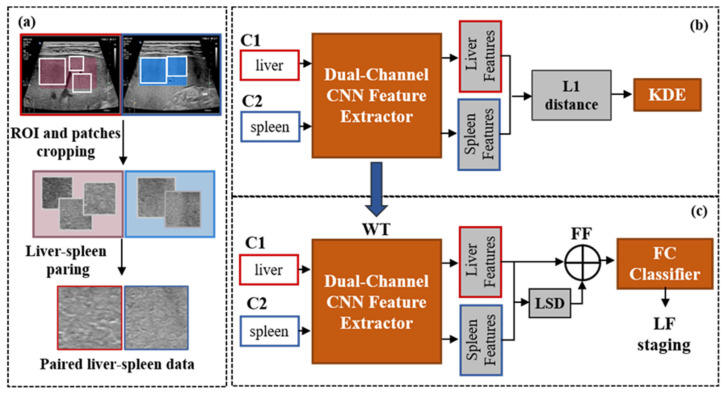
Overall algorithm scheme. Subfigure (**a**) shows the process of data preparation. Subfigure (**b**) shows the dual-channel feature extractor for liver and spleen feature extraction and the KDE method for LSD analysis. Subfigure (**c**) shows the dual-channel LF staging model. Abbreviations: ROI = region of interest, CNN = convolutional neural network, KDE = kernel density estimation, WT = weight transfer, LSD = liver–spleen difference, FC = fully connected, LF = liver fibrosis, FF = feature fusion.

The algorithm scheme is shown in Figure 4, and the details of each part are given in the following sections. Figure 4a shows the process of data preparation, during which the ROIs of the liver and spleen images were outlined by an experienced US doctor and the patches were cropped based on the ROI area. For every patient, one liver and one spleen patch was combined as a liver–spleen image pair (LS-pair) to train the dual-channel LF staging model. There were two stages in the training and validation process of the network. In the first stage, the Siamese feature extractor was trained to measure the L1 distance between the liver and the spleen as the value of the LSD, and the LSD value distributions under different degrees of LF were analyzed on the validation data using kernel density estimation (KDE), as shown in Figure 4b. In the second stage, the pretrained weights from stage one were transferred into the Siamese feature extractor of the LF staging model, and the fully connected (FC) classifier of the model was trained with the fusion of the liver and LSD features for LF staging as shown in Figure 4c.

### 2.5. Hepatic and Splenic Feature Representation

**Figure 5:**
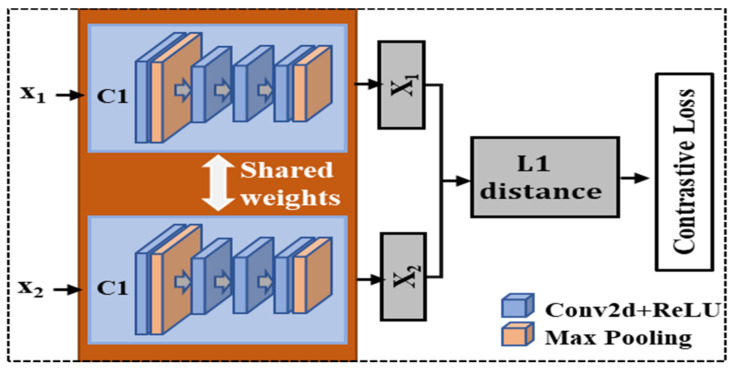
Structure of dual-channel CNN feature extractor.

The dual-channel feature extractor was constructed as a Siamese [31] structure with shared weights and trained with the contrastive cost function [32] as shown in Figure 5. Each channel is a convolutional neural network (CNN) and the structure of the CNN can be modified. The application of the CNN effectively avoided the complex artificial feature extraction and filtering steps of traditional algorithms, and the convolution operation transformed the original image into a high-dimensional feature representation, which could highlight the essential features that are difficult to detect with naked eyes. Two channels of the CNN extracted the liver and spleen features simultaneously, and the LSD was represented using Formula (2). The contrastive cost function is of the following form:

(1) $$L=\frac{1}{2N}\overset{N}{\underset{n=1}{\sum }}\left(1-y\right)d^{2}+y\max{\left(margin-d,0\right)}^{2}$$

where *N* is the number of LS-pairs for training; *d* is the value of LSD calculated with Formula (3); margin is a distance threshold, which is set to 2 in our study, and y is the label of the input pair, which equals 0 (means a small difference between the LS-pair) or 1 (means a large difference). With the contrastive cost function, we trained the feature extractor to recognize the differences between the liver and spleen US images of patients with different degrees of LF.

(2) $$LSD=\left|X_{1}-X_{2}\right|$$

(3) $$d\left(x_{1},\;x_{2}\right)=\overset{n}{\underset{i=1}{\sum }}\left|X_{1i}-X_{2i}\right|$$

### 2.6. Kernel Density Estimation

Kernel density estimation (KDE) is commonly used to visualize the distribution of data [33]. It is a nonparametric estimation method that studies the distribution information from the data itself, without utilizing prior knowledge or attaching any assumptions to data distribution. The value of LSD is one-dimensional data, and we used the KDE method to analyze its distribution under different degrees of LF. In our study, the KDE was defined as follows:

(4) $$\hat{f}\left(x,h\right)=\frac{1}{N}\overset{N}{\underset{i=1}{\sum }}K_{h}\left(x-x_{i}\right)$$

where *N* is the total number of samples and *K* is the kernel function, and we used a Gaussian kernel function in this study as shown in Formula (5). Furthermore, h is the hyperparameter bandwidth, which controls the relative weight of each sample by influencing the value of the independent variable in the kernel function as shown in Formula (6). In this study, we chose an adaptive method that determines the bandwidth based on the number of samples.

(5) $$K\left(x\right)=\frac{1}{\sqrt{2\pi }}exp\left(-\frac{x^{2}}{2}\right)$$

(6) $$K_{h}\left(x\right)=\frac{1}{h}K\left(\frac{x}{h}\right)$$

### 2.7. Weight Transfer and Feature Fusion

**Figure 6:**
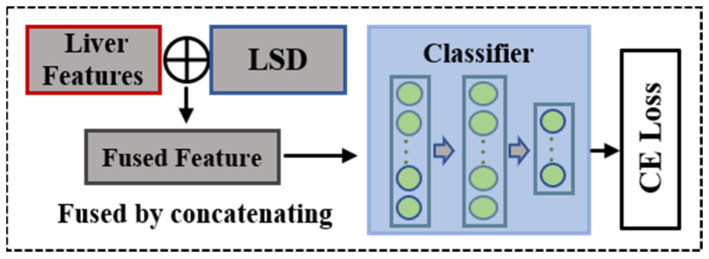
Feature fusion for FC classifier training.

To improve the performance of liver fibrosis staging by adding the LSD, we constructed an LF staging model that was composed of a dual-channel feature extractor and a fully connected (FC) classifier as shown in Figure 4c. During the feature extraction stage, we transferred the weights pretrained in the LSD measuring process shown in Figure 4b into the new model as a feature extractor. The features of the liver and the LSD were fused by concatenation as shown in Figure 6 to train the classifier with the cross-entropy (CE) cost function for LF staging. The CE cost function is of the following form:

(7) $$L=-\frac{1}{N}\overset{N}{\underset{i=1}{\sum }}\overset{M}{\underset{c=1}{\sum }}y_{ic}log\left(p_{ic}\right)$$

where *N* is the number of training samples, *M* is the number of categories, *y_ic_* is a symbolic function that equals 1 if sample *i* belongs to category *c* or 0 otherwise, and *p_ic_* is the prediction probability of sample *i* belonging to category *c*.

## 3. Results

We analyzed the correlation between the LSD value and the degree of fibrosis and combined the LSD with liver features to improve the performance of automated LF staging model. 

### 3.1. LSD Analysis

**Figure 7:**
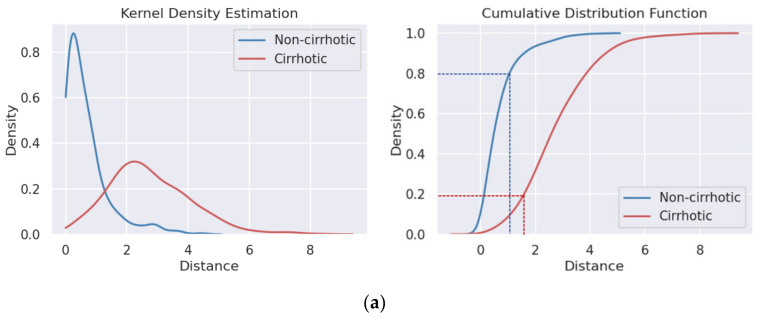

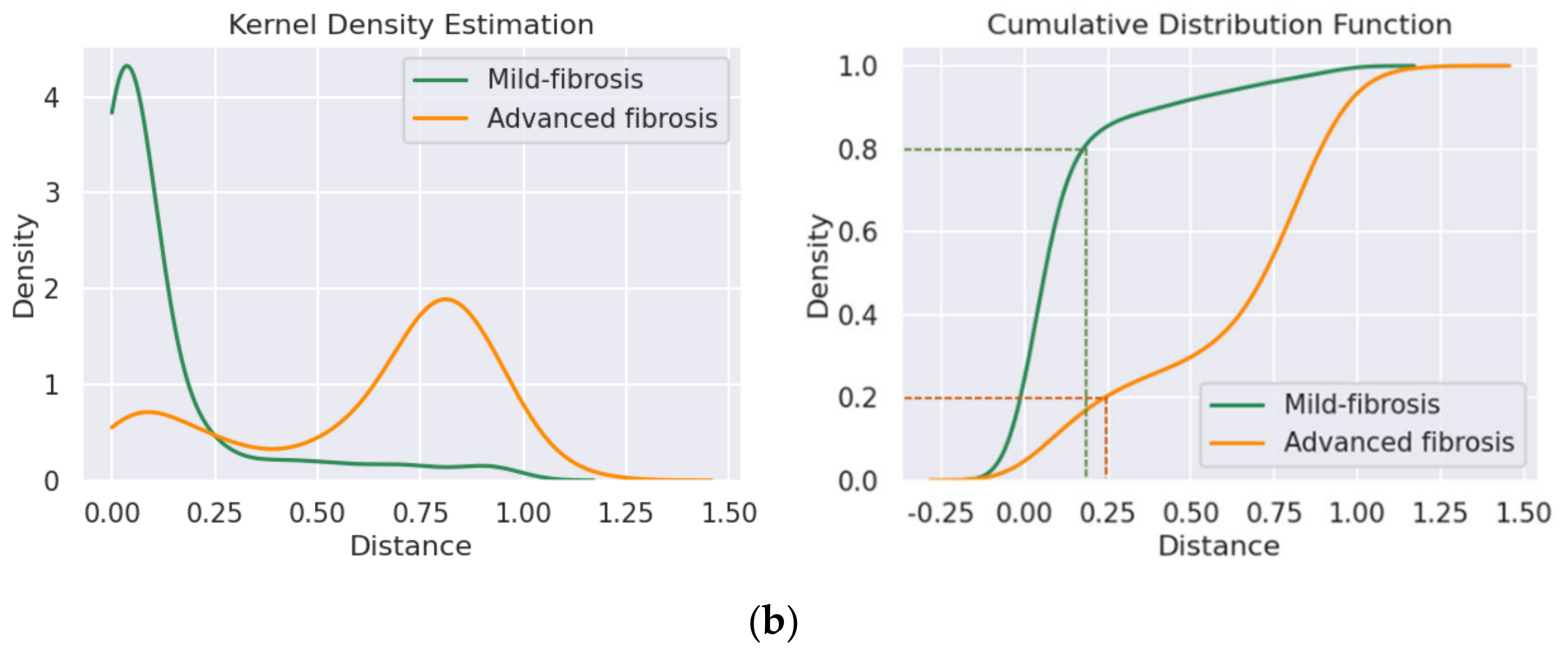
Kernel density estimation (KDE, **left**) and cumulative distribution function (CDF, **right**). (**a**) Noncirrhotic vs. cirrhotic. (**b**) Mild fibrosis vs. advanced fibrosis.

To analyze the LSD, we used KDE to estimate the distribution of LSD values under different degrees of LF and plotted the cumulative distribution function (CDF) for the comparison of important quantile statistics. In the KDE diagram, the abscissa represents the L1 distance of each liver–spleen image pair (i.e., the LSD value), and the ordinate represents the density of a distance value in the corresponding classes. The distribution of LSD values under different degrees of LF was obviously discrepant as shown in Figure 7; it shows that the higher the degree of fibrosis, the greater the distance overall, which means the greater the distinction between the liver and spleen US image signs. In the CDF diagram, the ordinate means the cumulative probability of distance. 

Figure 7a shows the KDE and CDF of a cirrhosis diagnosis; it shows the LSD value distributions under noncirrhotic (S0–S3) and cirrhotic (S4) cases. The average and two representative quantiles of LSD values from the CDF were extracted and are shown in Table 3. First of all, the average of the LSD value in cirrhotic cases was 2.74, which is much larger than that of noncirrhotic cases, 0.67. The 80% quantile of noncirrhotic cases was 0.98, which means that 80% of noncirrhotic cases had LSD values smaller than 0.98. Meanwhile, for cirrhotic cases, the 20% quantile of 1.67 shows that 80% of cirrhotic cases had LSD values larger than 1.67. From the LSD values’ distribution, we concluded that the difference between the liver and spleen was positively correlated with the degree of LF. Furthermore, the same conclusion can be reached from the analysis between mild-fibrosis (S0–S2) and advanced fibrosis (S3) as shown in Figure 7b.

**Figure 8:**
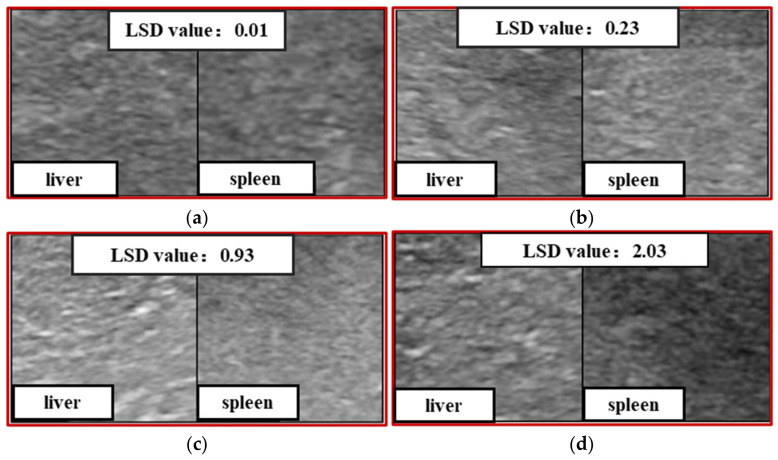
Examples of LSD values measured in cases with different degrees of fibrosis (S1–S4 means liver fibrosis stages 1–4, which were determined though liver biopsy by pathologists). (**a**) S0/S1. (**b**) S2. (**c**) S3. (**d**) S4.

Figure 8 shows example cases with different degrees of fibrosis, in which the LSD values were measured as the L1 distance by the dual-channel Siamese feature extractor. The comparison of liver and spleen US images from the same patient is helpful to highlight the fibrous changes in the liver parenchyma, and it indicates that the US splenic information has auxiliary values in US-image-based LF staging.

### 3.2. LF Staging

**Figure 9:**
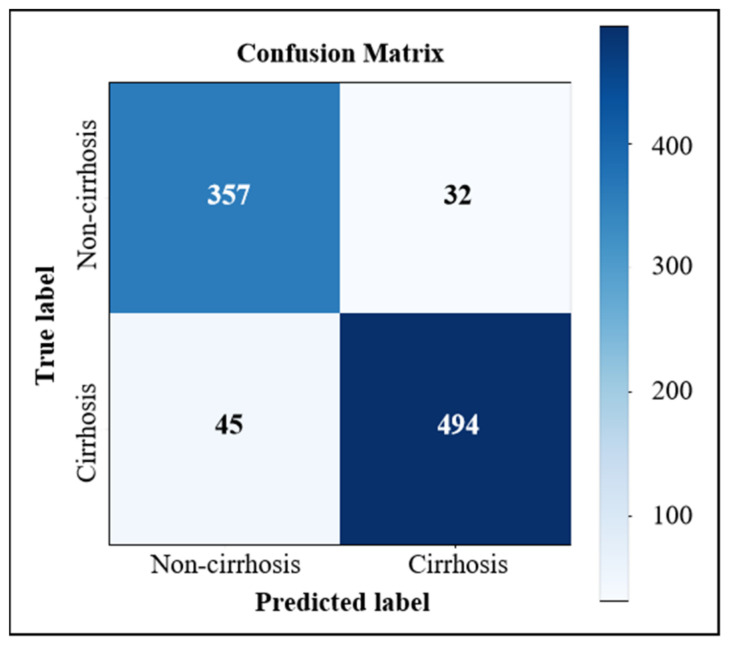
Confusion matrix of cirrhosis diagnosis based on dual-channel AlexNet.

**Figure 10:**
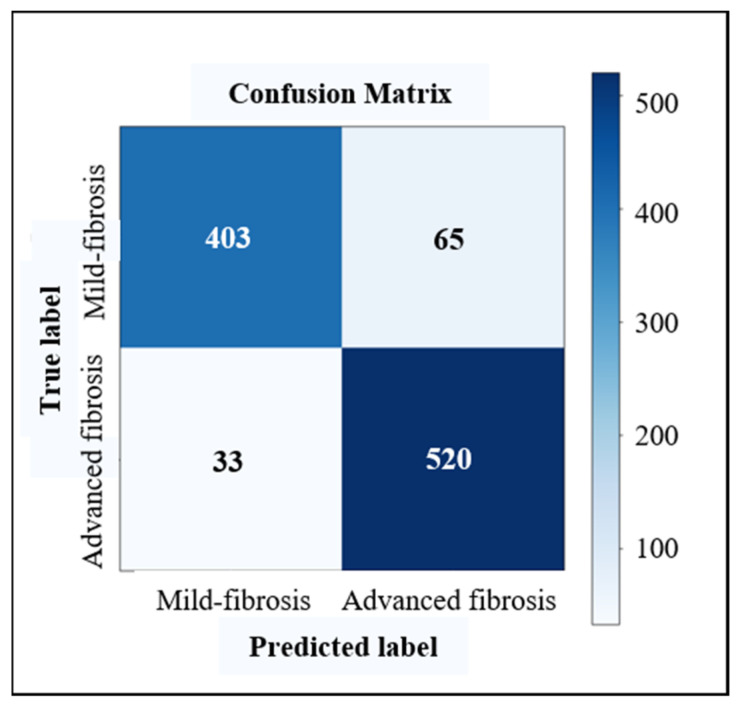
Confusion matrix of advanced fibrosis diagnosis based on dual-channel AlexNet.

**Figure 11:**
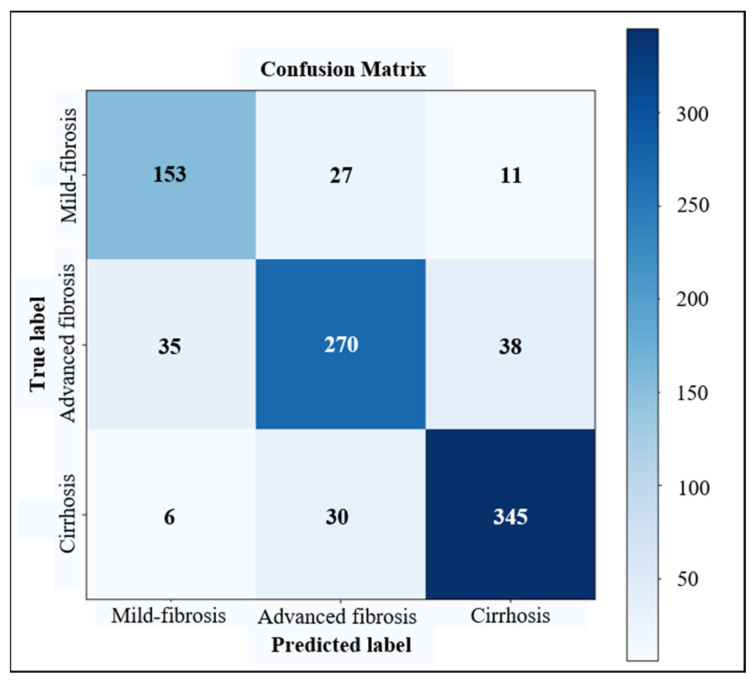
Confusion matrix of LF multi-staging based on dual-channel AlexNet.

In the task of fibrosis staging, we used the confusion matrix to record the predictions on a validating dataset. For instance, the confusion matrices for cirrhosis diagnosis, advanced fibrosis diagnosis and LF multi-staging based on the dual-channel AlexNet are shown in Figure 9, Figure 10 and Figure 11, respectively. According to the confusion matrix, the classification accuracy (acc), precision (p), recall (r) and f1-score (the harmonic average of p and r) were calculated using Formulas (8)–(11) as the evaluation indicators of the classification model. For the multi-classification task, the macro-averages of each indicator were taken as the indices.

(8) $$acc=\frac{TP+TN}{TP+FP+FN+TN}$$

(9) $$p=\frac{TP}{TP+FP}$$

(10) $$r=\frac{TP}{TP+FN}$$

(11) $$f1=\frac{2*p*r}{P+r}$$

We conducted experiments based on three commonly used deep CNN models in classification tasks (i.e., AlexNet, VGG16, GoogLeNet), using liver images to train ordinary models and LS-pairs to train the dual-channel staging models, respectively, and the results of cirrhosis identification (S0–3 vs. S4), advanced fibrosis diagnosis (S0–2 vs. S3–4) and three stages of fibrosis staging (S0–2 vs. S3 vs. S4) are shown in Table 4. Dual-channel LF staging models trained with LS-pairs performed better than ordinary classification models trained with liver images only, and the dual-channel staging model constructed with the feature extractor of AlexNet showed optimal overall performance.

For cirrhosis recognition and advanced fibrosis diagnosis, the most important clinical indicators are precision and recall, especially recall, which represents the sensitivity of identifying cirrhosis. In our study, the precision and recall greatly improved for dual-channel models trained with LS-pairs. The proposed dual-channel AlexNet improved the precision and sensitivity of a cirrhosis diagnosis to 93.92% and 91.65%, respectively, which is about 8% higher than that of the general model. The sensitivity of the advanced fibrosis diagnosis was 15% higher, which means the percentage of missed diagnoses decreased greatly. For LF multi-staging, the accuracy of classification improved 3–5% with dual-channel models. These results indicate that adding US splenic information is helpful in improving the performance of US-image-based LF staging models.

There were different numbers of liver and spleen image pairs for each patient. For a cirrhosis diagnosis, there were 928 pairs of validation images: 389 and 539 pairs of S0–S3 and S4, respectively. To evaluate the diagnostic consistency of our method on cirrhosis diagnosis, we input validation patients into the trained model case by case. For each patient, we defined four situations as follows: (1) 100% consistency (i.e., all pairs were correctly predicted); (2) 70%~99% consistency (i.e., 70%~99% pairs were correctly predicted); (3) 30%~69% consistency and (4) 0%~29% consistency. For situation (1) and (2), we considered the patient correctly diagnosed. Situation (3) meant that the patient was difficult to define, and further diagnosis was needed. Situation (4) meant misdiagnosis. There were 30 cases of noncirrhotic patients (S0–S3) and 30 cases of cirrhotic patients (S4) for evaluation, and the statistical results are shown in Table 5. This table shows that, for 75% of the validation cases, all pairs of liver and spleen images produced consistent and correct results in each case, and for 81.67% of validation cases, more than 70% of pairs in each case produced consistent and correct results. On a patient-by-patient basis, the proposed cirrhosis diagnosis model reached an 81.67% accuracy and a 6.67% misdiagnosis rate. Moreover, 11.66% of patients were defined as indeterminate and further examination was required.

## 4. Discussion

Liver fibrosis often presents a coarse echotexture in ultrasound images due to hyperplasia of the fibrous tissue. While performing an ultrasonic examination, clinicians always adjust the imaging settings such as gain, dynamic range, focus, transmit frequency and speckle reduction according to the differences in obesity and the fibrosis degree of each patient. Nevertheless, the imaging parameters are subjectively optimized by ultrasound doctors, so the image quality is quite dependent on the experience and operation skills of the doctors and individual differences and noise are also important factors affecting image homogeneity. Variations in the image quality may bring confusion to image-based diagnosis. Echotexture changes in the liver parenchyma will cause differences in hepatic and splenic US image signs in patients (LSDs). In this study, we proposed a dual-channel Siamese feature extractor to quantify the LSD with the L1 distance, and the KDE analysis showed that the higher the degree of LF in patient, the greater the LSD value. We fused the LSD with hepatic features for LF staging and achieved better results than methods based on liver features only. 

In recent years, LF staging algorithms based on US examination and machine learning often require the liver stiffness, clinical parameters or serum markers of patients. However, a complex data collection process is not conducive to the rapid staging of LF. Taking the diagnosis of cirrhosis as an example, Table 6 presents the performance of some related algorithms in cirrhosis diagnoses in recent years. Compared with related studies, our proposed method did not require complex parameters of patients or complicated manual feature extraction and reached a competitive performance in cirrhosis diagnosis.

Ultrasound experts usually consider many more parameters of patients than the US image signs while making a clinical diagnosis of liver fibrosis; however, with the advantages (such as speed, simplicity and low cost) of ultrasound examination, we hope the proposed model can be applied in these two aspects: (1) Identification of patients at risk of serious liver fibrosis during screening and referral to a liver specialist for timely treatment, to avoid developing into life-threating disease. (2) Follow-up of patients with cirrhosis and advanced fibrosis during treatment to provide diagnosis reference for doctors, monitor the therapeutic efficacy of patients and avoid abnormalities.

As a pilot study, there still are some limitations to this work. (1) The unitary type of the illness. All patients included in this study had different degrees of liver fibrosis due to hepatitis B, and there was no case of fibrosis caused by another kind of CLD. (2) The comparison between the liver and spleen was based on the premise that the patients did not have any splenic diseases, otherwise the diagnosis result may have been affected. In future research, it is necessary to establish a larger dataset with various types of CLDs to improve the robustness and generalization of the proposed method and make better use of splenic information to improve the performance of US-image-based automated LF staging models. 

## 5. Conclusions

This study proposed a two-stage Siamese network combining hepatic and splenic US images for noninvasive liver fibrosis staging. Stage one used a Siamese network to analyze the difference between liver and spleen US features (i.e., LSD) in patients with different degrees of fibrosis, and the results indicated that the higher the degree of liver fibrosis, the greater the LSD in most patients. Stage two combined the LSD with liver features for liver fibrosis staging and achieved better performance than general DL classification models. The study shows great potential of splenic information in US-images-based liver fibrosis staging.

## Figures and Tables

**Table 1:** Demographics of patients and the clinical description of Scheuer scoring system (‘-’ means not involved, and grades of fibrosis are determined according to histology).

Characteristics	Value	Scheuer Scoring System Description
Number of patients	286	-
Age	52.4 [40.30–61]	-
Sex		
Male	216 (75.42%)	-
Female	70 (24.58%)	-
BMI (kg/m^2^)	23.39 [20.80–26]	-
Grade		
0	14 (4.90%)	None
1	29 (10.14%)	Enlarged, fibrotic portal tracts
2	60 (20.98%)	Periportal or portal–portal septa but intact architecture
3	78 (27.27%)	Fibrosis with architectural distortion but no obvious cirrhosis
4	105 (36.71%)	Probable or definite cirrhosis

**Table 2:** The corresponding relationship between the US image signs and the stages of LF in patients with hepatitis B (reference [29]).

Grade	Ultrasound Image Signs
0	Normal liver size, smooth liver capsule, **homogeneous or slightly rough echo of liver parenchyma,** clear vascular orientation, without splenomegaly.
1	Normal liver size, smooth liver capsule, **rough echo of liver parenchyma,** clear vascular orientation, without splenomegaly.
2	Moderate liver size, smooth liver capsule, **significantly rough and enhanced echo of liver parenchyma, visible brightened linear structure shown as “strip pattern”,** still clear vascular orientation, without splenomegaly.
3	Moderate or slightly smaller liver size, unsmooth liver capsule, **significantly rough and enhanced echo of liver parenchyma with uneven distribution, with or without hyperplasia nodules,** vague vascular terminals, with or without splenomegaly.
4	Smaller liver size, wavy liver capsule, **significantly rough and unevenly enhanced echo of liver parenchyma, visible patchy enhancement, with or without nodules,** variant vascular stenosis, with or without splenomegaly.

**Table 3:** Representative quantiles of CDF. “-” represents meaningless data.

Subfigure	Class	Average	20% Quantile	80% Quantile
Figure 7a	Noncirrhotic	0.67	-	0.98
	Cirrhotic	2.74	1.67	-
Figure 7b	Mild fibrosis	0.12	-	0.14
	Advanced fibrosis	0.62	0.22	-

**Table 4:** Results of fibrosis staging.

Diagnostic Objectives	Feature Extractors	Training Data	Acc (%)	(Macro-) Precision (%)	(Macro-) Recall (%)	(Macro-) f1 (%)
S0–3 vs. S4	GoogLeNet	Liver	81.45	80.25	72.22	76.02
		LS-pairs	79.53	80.14	86.09	83.01
	VGG16	Liver	86.88	83.52	84.44	83.98
		LS-pairs	87.28	91.85	85.71	88.68
	AlexNet	Liver	87.33	85.23	83.33	84.27
		LS-pairs	91.70	93.92	91.65	92.76
S0–2 vs. S3–4	GoogLeNet	Liver	78.73	82.09	61.11	70.06
		LS-pairs	75.18	82.34	77.14	79.65
	VGG16	Liver	82.81	85.14	70.00	76.83
		LS-pairs	87.91	84.02	94.74	89.06
	AlexNet	Liver	85.52	84.52	78.89	81.61
		LS-pairs	90.40	88.89	94.03	91.39
S0–2 vs. S3 vs. S4	GoogLeNet	Liver	61.36	55.94	58.83	54.34
		LS-pairs	64.04	61.74	59.49	57.79
	VGG16	Liver	72.27	71.56	71.71	71.51
		LS-pairs	77.81	75.80	76.40	75.21
	AlexNet	Liver	77.25	78.57	76.82	76.95
		LS-pairs	83.93	83.00	83.12	83.04

**Table 5:** Predictive consistency of liver cirrhosis diagnosis model.

Degrees	Validation Cases	Consistency (Cases)			
		100%	70~99%	30~69%	0~29%
S0–S3	30	26 (86.66%)	0	2 (6.67%)	2 (6.67%)
S4	30	19 (63.33)	4 (13.33%)	5 (16.67%)	2 (6.67%)
Total	60	45 (75.00%)	4 (6.67%)	7 (11.66%)	4 (6.67%)

**Table 6:** Performance of related algorithms on cirrhosis diagnosis (“-” means data not mentioned).

Data	Methods	Results			
		Acc	P	R	AUROC
[21] 230 patients’ US radiofrequency data	One-dimensional CNN	91.67%	-	88.89%	0.934
[23] 187 patients’ US images	a. EfficientNet b. SVM + 637 manually extracted features	a. 80% b. -	-	a. 99% b. -	a. 0.83 b. 0.96
[24] 681 patients’ US images	VGGNet	-	-	-	0.948
[26] 508 patients’ US images	Multi-scale texture network (MSTNet)			87.8%	0.89
[27] 3446 patients’ US images	VGG16	0.902	-	94.1%	0.901
[34] 466 patients a. gray-scale modality + elastogram modality images b. gray-scale modality images + liver stiffness measurement	Inception-V3	-	-	a. 90.1% b. 89.0%	a. 0.950 b. 0.937
**Our method**		91.70%	93.92%	92.77%	0.97

## Data Availability

The data presented in this study are available on request from the corresponding author. The data are not publicly available due to ethical restrictions.
